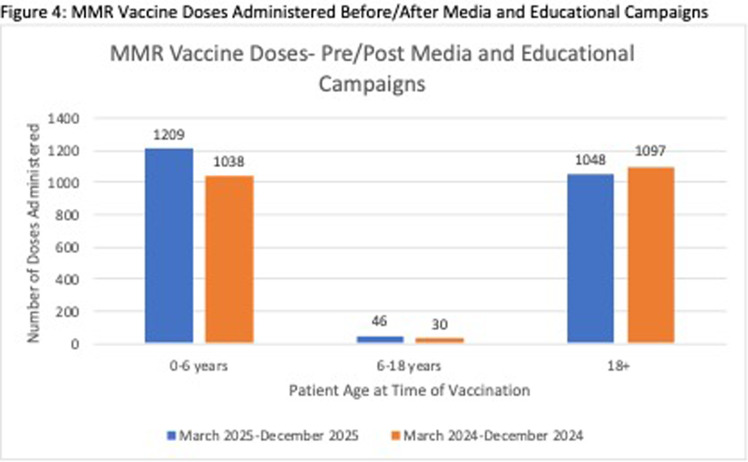# 300 Hospital-Onset Sepsis Events: Preventability and Opportunities for Improvement

**DOI:** 10.1017/ash.2026.10452

**Published:** 2026-06-23

**Authors:** Nicholas Van Sickels, Takaaki Kobayashi, Rachel Howard, Hollie Sands, Kathleen Winter, Bethany Hodge, Derek Forster, Sarah Cotner

**Affiliations:** 1 University of Kentucky; 2 UK Healthcare; 3 Lexington-Fayette County Health Department; 4 Kentucky Department for Public Health; 5 University of Kentucky School of Medicine; 6 UK HealthCare

## Abstract

**Background:** The 2025 Measles outbreak has affected over 1900 people across the United States. Close collaborations between public health entities and training institutions can increase public knowledge and trust, promote vaccination, optimize options for testing, and reduce exposures. **Methods:** The Lexington Fayette County Health Department (LFCHD), Kentucky Department for Public Health (KDPH), and the University of Kentucky (UKY) partnered in February 2025 to develop a comprehensive plan to address measles in Kentucky. Priorities for the partnership were: community and provider education, delineation of roles/responsibilities between the health department and UKY, streamlined pathways for testing and post-exposure management, and centralized communication. Standardized educational sessions were developed and deployed widely to outline testing and post-exposure prophylaxis (PEP) resources. Infection Prevention and Control (IPAC) physicians triaged all testing requests in partnership with the health department using CDC criteria for testing. Measles, Mumps, and Rubella (MMR) vaccination campaigns were conducted, focusing on prevention and post-exposure management. For healthcare workers (HCW) at UKY, Epic® electronic health record (EHR) templates and order sets outlining CDC and KDPH algorithms were developed and distributed widely. **Results:** From March through October 2025, the standardized educational talk was given to 398 public health and medical professionals (Table 1). Six public health and healthcare systems adapted the public health-academic center partnership and protocol to their centers (Table 2). Joint media outreach and education resulted in 32 stories across Kentucky and Tennessee. Three Epic® EHR templates were developed and deployed widely (Figures 1-3). IPAC received and triaged approximately 50 calls/EMR messages from HCW about measles testing, resulting in 4 tests performed at the KDPH State Lab. One test returned positive and there were zero healthcare worker exposures. Immune globulin for PEP was offered to 2 exposed patients, though they declined to receive it (Table 2). MMR vaccinations increased by 16% and 53% in the 0-6 year and 6–18-year age groups, respectively from 2024 to 2025, during the same time periods. MMR vaccination in persons over 18 years decreased slightly (4.5%) (Figure 4). **Conclusions:** Combining the infrastructure, knowledge, and resources of a state and local public health systems with an academic medical center’s training and community trust can increase measles awareness, vaccination, and optimize resources during an outbreak.

**Figure:**
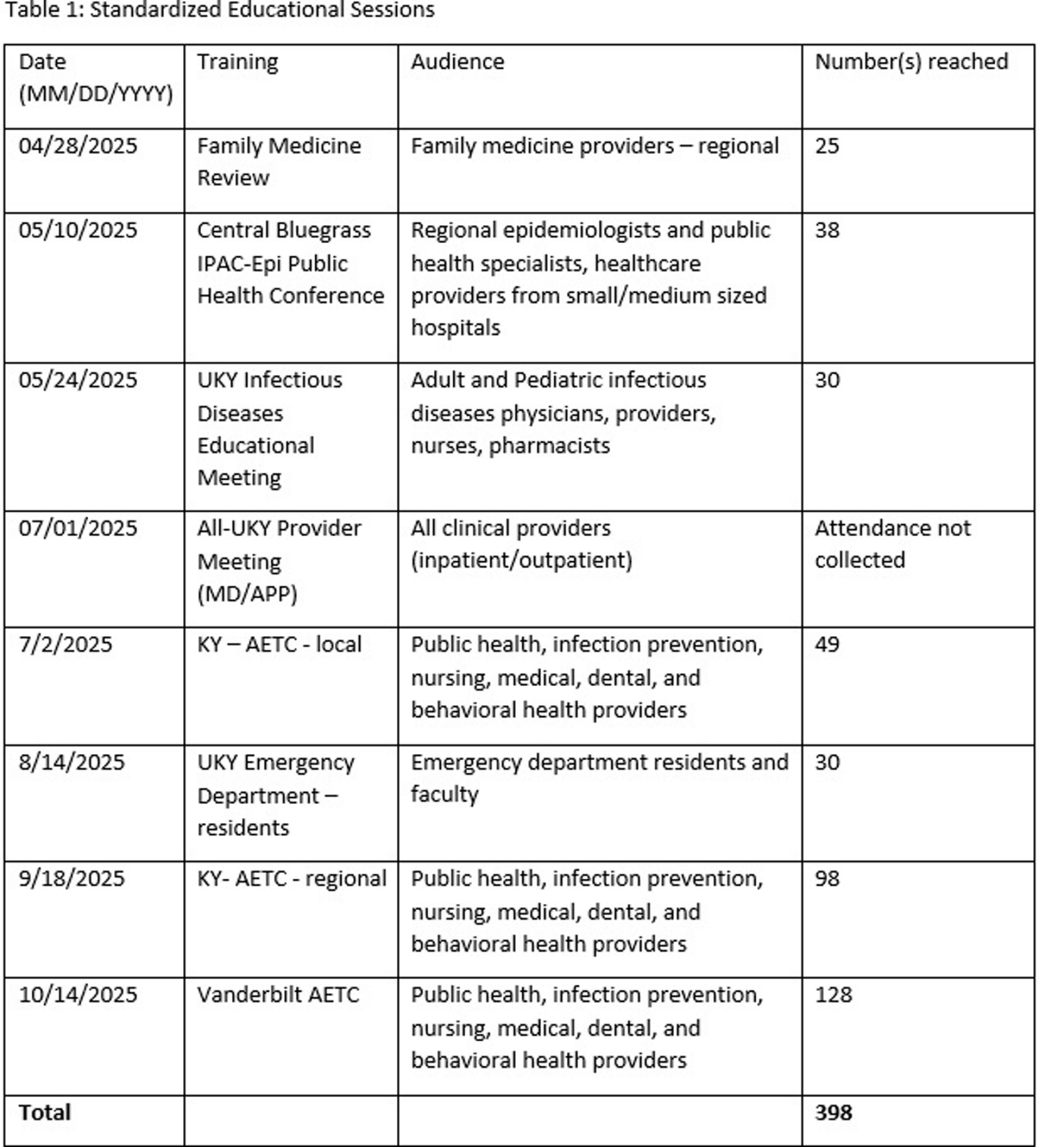

**Figure:**
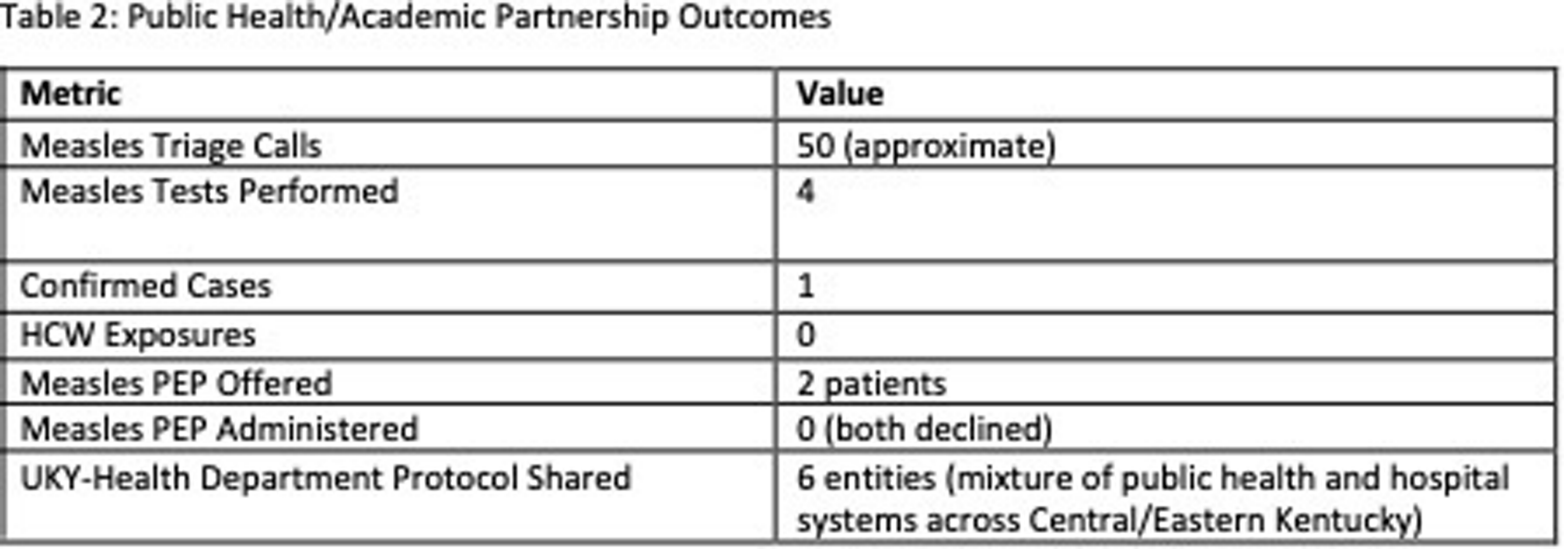

**Figure:**
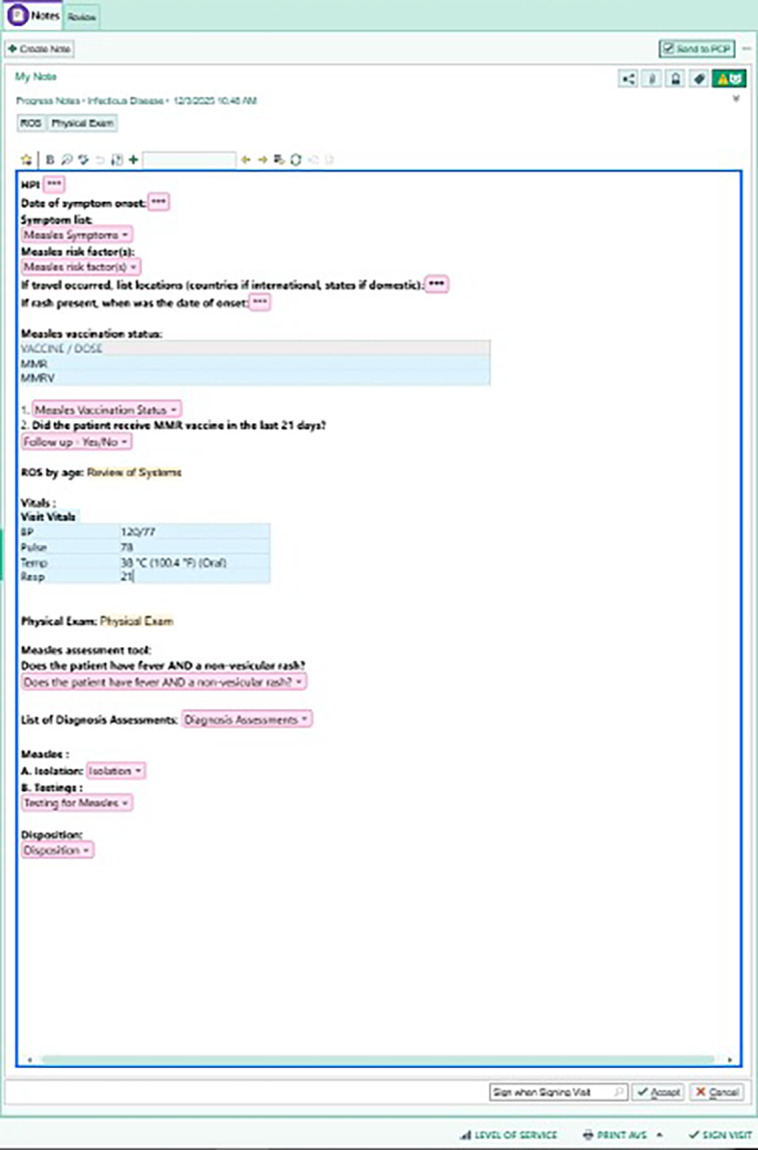

**Figure:**
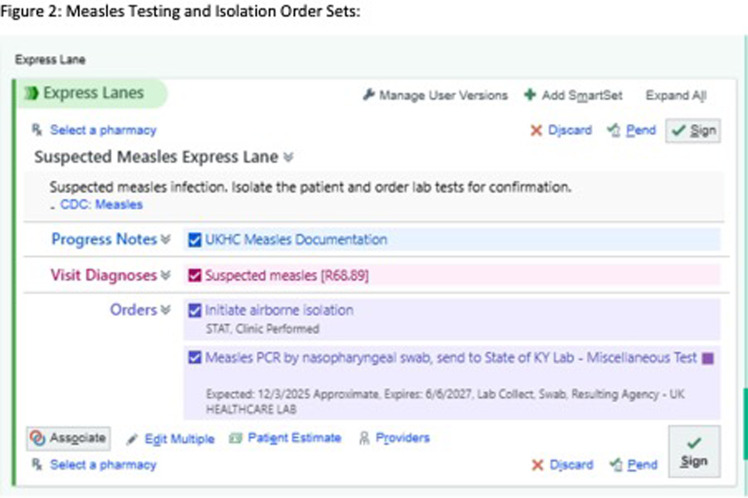

**Figure:**
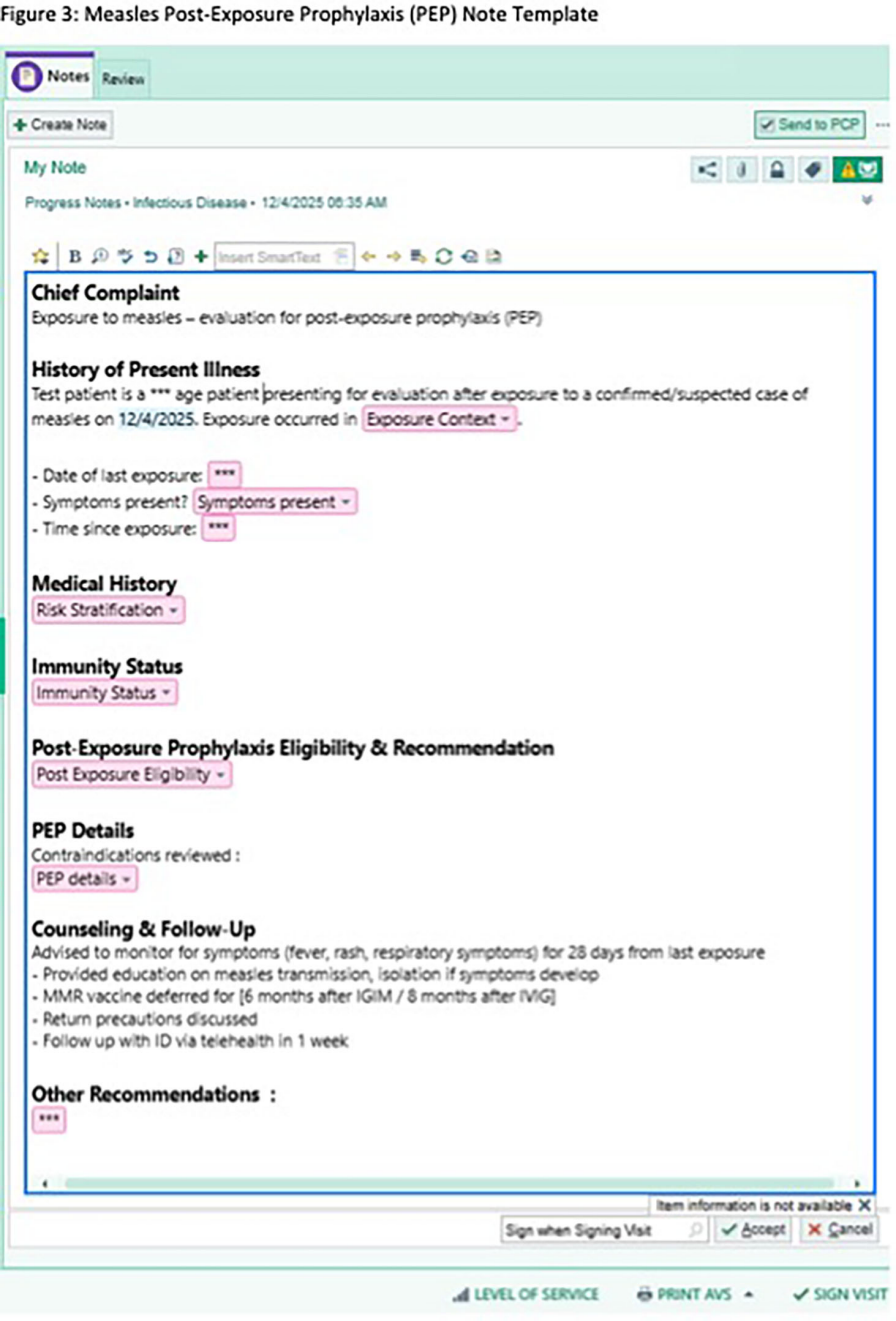

**Figure:**